# *Saccharomyces cerevisiae* Bat1 and Bat2 Aminotransferases Have Functionally Diverged from the Ancestral-Like *Kluyveromyces lactis* Orthologous Enzyme

**DOI:** 10.1371/journal.pone.0016099

**Published:** 2011-01-18

**Authors:** Maritrini Colón, Fabiola Hernández, Karla López, Héctor Quezada, James González, Geovani López, Cristina Aranda, Alicia González

**Affiliations:** 1 Departamento de Bioquímica y Biología Estructural, Instituto de Fisiología Celular, Universidad Nacional Autónoma de México, México City, México; 2 Departamento de Bioquímica, Instituto Nacional de Cardiología, México City, México; University College Dublin, Ireland

## Abstract

**Background:**

Gene duplication is a key evolutionary mechanism providing material for the generation of genes with new or modified functions. The fate of duplicated gene copies has been amply discussed and several models have been put forward to account for duplicate conservation. The specialization model considers that duplication of a bifunctional ancestral gene could result in the preservation of both copies through subfunctionalization, resulting in the distribution of the two ancestral functions between the gene duplicates. Here we investigate whether the presumed bifunctional character displayed by the single branched chain amino acid aminotransferase present in *K. lactis* has been distributed in the two paralogous genes present in *S. cerevisiae*, and whether this conservation has impacted *S. cerevisiae* metabolism.

**Principal Findings:**

Our results show that the *Kl*Bat1 orthologous BCAT is a bifunctional enzyme, which participates in the biosynthesis and catabolism of branched chain aminoacids (BCAAs). This dual role has been distributed in *S. cerevisiae* Bat1 and Bat2 paralogous proteins, supporting the specialization model posed to explain the evolution of gene duplications. *BAT1* is highly expressed under biosynthetic conditions, while *BAT2* expression is highest under catabolic conditions. Bat1 and Bat2 differential relocalization has favored their physiological function, since biosynthetic precursors are generated in the mitochondria (Bat1), while catabolic substrates are accumulated in the cytosol (Bat2). Under respiratory conditions, in the presence of ammonium and BCAAs the *bat1Δ bat2Δ* double mutant shows impaired growth, indicating that Bat1 and Bat2 could play redundant roles. In *K. lactis* wild type growth is independent of BCAA degradation, since a *Klbat1Δ* mutant grows under this condition.

**Conclusions:**

Our study shows that *BAT1* and *BAT2* differential expression and subcellular relocalization has resulted in the distribution of the biosynthetic and catabolic roles of the ancestral BCAT in two isozymes improving BCAAs metabolism and constituting an adaptation to facultative metabolism.

## Introduction

It is accepted that *Saccharomyces cerevisiae* genome arose from complete duplication of eight ancestral chromosomes; functionally normal ploidy was recovered due to the massive loss of 90% of duplicated genes. Analysis of the complete yeast genome sequence identified several interchromosomal duplicated regions [1], [2] which constitute the molecular evidence of an ancient duplication of the entire yeast genome [3]. Gene duplication and the subsequent divergence of paralogous pairs play an important role in the evolution of novel gene functions. Several models have been proposed to account for the emergence, maintenance and evolution of gene copies. It has been shown that diversification of paralogous genes whose products are strictly involved in amino acid biosynthesis has led to functional diversification such that retention of both copies is needed to fulfill the function carried out by the original gene [4]–[6], thus supporting the duplication-degeneration-complementation model proposed by Force *et al*. [7]. The specialization or escape from adaptive conflict posed by Hughes [8] considers that if the original gene was performing two functions, that could not be independently improved, after duplication each gene copy could be driven by positive selection to improve one of the two functions. Aminotransferases constitute an interesting model to study diversification of paralogous genes carrying out two functions, both of which are needed to warrant metabolite provision, and which cannot be differentially improved, since aminotransferases constitute biosynthetic and catabolic pathways whose opposed action relies on a single catalytic site. Furthermore, metabolite provision through the action of aminotransferases, is necessary when yeast is grown in either fermentable or non-fermentable carbon sources and thus, functional diversification of aminotransferase-encoding paralogous genes could play a fundamental role in the adaptation to facultative metabolism.

In the yeast *Saccharomyces cerevisiae*, the last step in the biosynthesis and the first step in the catabolism of branched chain amino acids (BCAAs), is achieved through the action of the branched chain aminotransferases (BCATs) encoded by the paralogous pair *BAT1* and *BAT2*, which form part of a duplicated chromosomal block generated from the Whole Genome Duplication (WGD) event [2], [3] (http://www.gen.tcd.ie/~khwolfe/yeast/nova/index.html). An additional inspection using the Yeast Gene Order Browser (http://wolfe.gen.tcd.ie/ygob/) also suggests that *BAT1*/*BAT2* could be in a duplicated block. This evidence points to the origin of the *BAT1-BAT2* duplicated gene pair as part of the WGD duplication event rather than to an isolated gene duplication phenomenon. These enzymes catalyze the transfer of amino groups between the amino acids valine, leucine and isoleucine and their corresponding α-ketoacids, the biosynthetic precursors of fusel alcohols, which influence the aroma and flavor of yeast derived fermentation products such as beer and bread [9], [10], and which have been recently found to regulate translation initiation by inhibiting eIF2B [11].

The lineage which gave rise to *Kluyveromyces lactis* (*K. lactis*) diverged before the WGD event, therefore, *K. lactis* genome does not harbor the duplication blocks present in *S. cerevisiae* [3]. In *K. lactis* the gene *KlBAT1* constitutes, the unique orthologue of the *S. cerevisiae BAT1* and *BAT2* paralogous gene pair encoding a branched chain aminotransferase (*Kl*Bat1). We have undertaken the study of the functional role played by *Kl*Bat1, Bat1 and Bat2, in order to understand whether the role played by the ancestral-type enzyme has been conserved in Bat1 and Bat2 resulting in redundant function or whether it has been distributed between these two enzymes resulting in diversification.

*Kl*Bat1 encoded protein is constituted by 407 amino acid residues and as well as Bat1 it bears an amino terminal signal peptide which could direct its mitochondrial localization. It shares 82% amino acid identity with Bat1 and 79% with Bat2. *BAT1* encodes a 393 amino acid residues mitochondrial protein, while the cytosolic Bat2 is composed of 376 residues; these two enzymes show 81% identity. Previous results from other laboratories have shown that on glucose-containing media, *BAT1* single deletion impaired neither cell growth nor fusel alcohol production; however, drastic effects in fusel alcohol production were observed in a *bat2Δ* deletion mutant. Deletion of both genes resulted in branched chain amino acid auxotrophy, severe growth retardation and diminished fusel alcohol production [12]. The fact that the enzymes involved in the biosynthesis of the BCAAs are mitochondrially located has led to the notion that in *S. cerevisiae*, the biosynthetic process is mainly carried out in the mitochondria. However, the fact that Bat1 and Bat2 are located in both compartments indicates that the last step in BCAAs biosynthesis can be carried out in either the mitochondria or the cytoplasm. Furthermore, for the leucine biosynthetic pathway, Leu1 and Leu2 have been only found in cytosol [13], [14] indicating that the conversion of α-ketoisovalerate to α-isocaproate the immediate precursor of leucine is carried out in the cytoplasm and further transported to the mitochondria so that the last step in leucine biosynthesis can be carried out in either the mitochondria or the cytoplasm, through the action of either Bat1 or Bat2. No analysis has been undertaken to determine the compartment in which BCAAs catabolism is carried out and the physiological role of differential Bat1 and Bat2 localization has not been analyzed.

Results presented in this paper support the specialization model posed by Hughes [8], showing that i) *K. lactis KlBAT1* codifies a presumed mitochondrial localized BCAT, which participates in both, the biosynthesis and catabolism of BCAAs, which is unable to complement *S. cerevisiae bat2Δ* mutants, and that ii) in *S. cerevisiae* biosynthetic and catabolic roles have been distributed in two paralogous genes. Bat1 is preferentially involved in BCAAs biosynthesis, while Bat2 function is determinant for BCAAs catabolism, indicating functional diversification. The specialization has been afforded through differential subcellular localization of the encoded products and divergent gene expression patterns, which is reflected in enzyme activity under various physiological conditions.

## Results

### The ancestor-like branched chain aminotransferase *Kl*Bat1 is a bifunctional biosynthetic and catabolic enzyme

A *Klbat1Δ* mutant incubated on glucose and ammonium, displayed valine, isoleucine and leucine (VIL) auxotrophy (Table 1). Wild type growth was only attained when the three BCAAs were simultaneously added to the growth medium. The *Klbat1Δ* mutant did not grow when branched chain amino acids were supplemented as sole nitrogen sources (Table 1), showing that this enzyme is also involved in BCAAs catabolism. These results indicate that no redundant pathways are involved in VIL biosynthesis and catabolism. As expected, *Klbat1Δ* transformants carrying the *KlBAT1* gene on a centromeric plasmid displayed wild type phenotype when grown on either ammonium-glucose or VIL-glucose (Table 1), indicating that *Kl*Bat1 is a bifunctional enzyme, which participates in VIL biosynthesis and catabolism.

**Table 1:** *Klbat1Δ* mutants are impaired in VIL biosynthesis and catabolism.

	Relative growth^a^ (%)		
	Glucose		
Strain	NH_4_^+^	NH_4_^+^ VIL^b^	VIL
*Kl*WT	100	100	100
CLA34 (*Klbat1Δ*)	0	96	0
*Kl*WT (pKD1)	100	100	100
*Kl*WT (pKD1 *KlBAT1*)	100	100	100
CLA34 (pKD1)	0	N. D.^c^	0
CLA34 (pkD1 *KlBAT1*)	100	N. D.	100

a Values are shown relative to growth rate of the wild type strain (0.12 h^−1^ and 0.13 h^−1^ on NH_4_^+^ and amino acids, respectively); and represent the means from three independent experiments (variation was always ≤10%).

b Amino acids were supplemented at a concentration of 150 mg/l, 100 mg/l or 30 mg/l of valine (V), leucine (L) or isoleucine (I) respectively.

c N. D. not determined.

### In *S. cerevisiae*, biosynthetic and catabolic roles of the branched chain aminotransferases have been differentially distributed in the *BAT1* and *BAT2*-encoded isozymes

**Figure 1:**
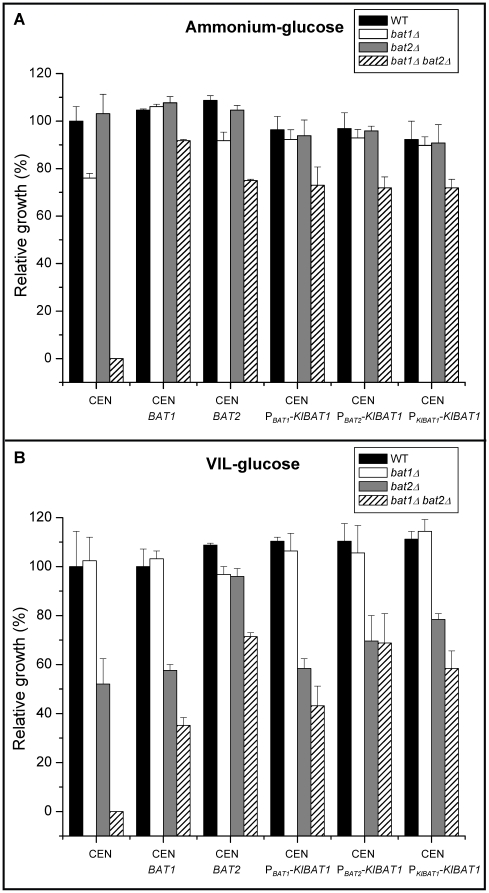
Growth phenotype of single and double mutants complemented with plasmids harboring *BAT*1, *BAT2* or *KlBAT1*. Wild type, *bat1Δ*, *bat2Δ* and *bat1Δ bat2Δ* strains were grown on ammonium-glucose (A) or VIL-glucose (B). Values are shown relative to growth rate of the wild type strain (0.20 h^−1^ and 0.13 h^−1^ on ammonium-glucose and VIL glucose respectively) and represent the mean of three independent experiments ± S. D. Cells were complemented with a centromeric plasmid (CEN) harboring *BAT1* (CEN *BAT1*), *BAT2* (CEN *BAT2*) or the *K. lactis* orthologous gene *KlBAT1* whose expression was driven by its own promoter (CEN P*_KlBAT1_-KlBAT1*) or by *BAT1* (CEN P*_BAT1_-KlBAT1*) or *BAT2* (CEN P*_BAT2_-KlBAT1*) promoters.

**Figure 2:**
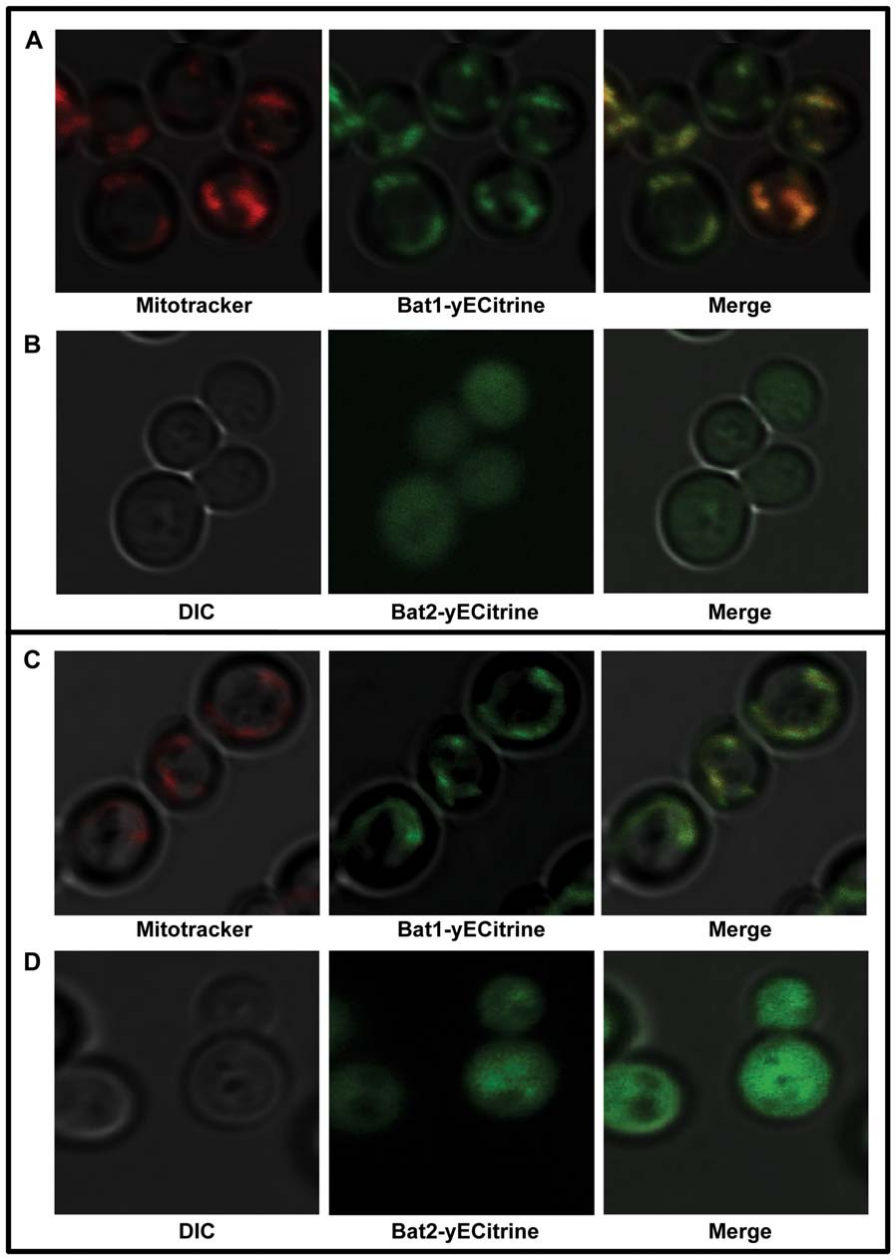
Bat1 is mitochondrially located, while Bat2 is cytoplasmic. Fluorescence images showing the subcellular localization of the paralogous proteins Bat1 and Bat2. Samples were taken from exponentially grown cultures of tagged mutants grown on glucose-ammonium (A, B) or on glucose-VIL (C, D). Mitochondrial localization of Bat1, the signal of the Bat1-yECitrine fusion co-localizes with mitotracker signal (A, C). Cytoplasmic localization of the Bat2-yECitrine fusion (B, D).

Single and double *bat1Δ* and *bat2Δ* mutants were constructed. As Table 2 and Figure 1A, CEN show, a double *bat1Δ bat2Δ* mutant displayed VIL auxotrophy when incubated on glucose and ammonium; wild type growth was attained when this strain was grown in the presence of the three BCAAs (Table 2). *BAT1* and *BAT2* were independently cloned on centromeric plasmids and used to transform the *bat1Δ bat2Δ* mutant. Transformants carrying *BAT1* recovered VIL prototrophy (Figure 1A, CEN *BAT1*), while those carrying *BAT2* showed a bradytrophyc phenotype (Figure 1A, CEN *BAT2*), indicating that Bat1 had a more efficient biosynthetic role than that exerted by Bat2. When cultured on ammonium-glucose, the single *bat1Δ* mutant showed a significantly decreased growth rate (69%), as compared to the wild type strain however, it attained wild type growth rates by the sole addition of valine to the growth medium (94%) (Table 2), or when complemented with a centromeric plasmid harboring *BAT1* (Figure 1A, CEN *vs.* CEN *BAT1*). *BAT2* did not complement *bat1Δ* growth deficiency (Figure 1A, CEN *BAT2*). These results indicate that Bat1 activity is indispensable to fulfill valine requirement and that Bat2 is unable to fully replace Bat1, suggesting functional diversification. Accordingly, the single *bat2Δ* mutant grew as well as the wild type on ammonium-glucose, with or without amino acids (Table 2), confirming that Bat1 completely fulfilled biosynthetic needs. Since it has been proposed that Bat2 is cytosolic, while Bat1 is mitochondrially located [9] it could be considered that the valine pool generated through Bat2 might not be efficiently transported to the mitochondria. In order to confirm *in vivo* enzyme localization, Bat1-yECitrine and Bat2-yECitrine tagged strains were constructed as described in Materials and Methods and subcellular localization was analyzed by confocal microscopy. As Figure 2 shows, Bat1 was found to be localized in mitochondria, while Bat2 was cytosolic, confirming previous observations [9]. It could thus be proposed that as mentioned above, the valine pool synthesized through Bat2 is not efficiently transported to the mitochondria or that valine synthesis through Bat2 is scarce leading to the observed valine braditrophy of a *bat1Δ* mutant.

**Table 2:** In *S. cerevisiae bat1Δ* mutant is impaired in VIL biosynthesis, while a *bat2Δ* mutant is mainly impaired in VIL catabolism.

	Relative growth^a^ (%)								
	Glucose								
Strain	NH_4_^+^	NH_4_^+^ V^b^	NH_4_^+^ I	NH_4_^+^ L	NH_4_^+^ VIL	V	I	L	VIL
CLA1-2 (WT)	100	100	100	100	100	100	100	100	100
CLA31 (*bat1Δ*)	69	94	65	65	100	97	88	78	100
CLA32 (*bat2Δ*)	100	95	93	100	100	61	52	78	70
CLA33 (*bat1Δ bat2Δ*)	0	0	0	0	91	0	0	0	0

a Values are shown relative to growth rate of the wild type strain (0.20 h^−1^ and 0.11 h^−1^ on NH_4_^+^ and amino acids, respectively); and represent the means from three independent experiments (variation was always ≤10%).

b Amino acids were supplemented at a concentration of 150 mg/l, 100 mg/l or 30 mg/l of valine (V), leucine (L) or isoleucine (I) respectively.

To analyze the role of Bat1 and Bat2 on VIL catabolism, *bat1Δ bat2Δ* double mutant and single mutants were grown on glucose in the presence of the three BCAAs as sole nitrogen source. Under these conditions, the wild type strain and the *bat1Δ* mutant showed higher growth rates than those observed in the double and *bat2Δ* mutants indicating a catabolic role for Bat2 (Table 2 and Figure 1B, CEN). On VIL-glucose *bat2Δ* mutant was only able to achieve 70% of the growth rate displayed by the wild type strain, suggesting that Bat2-dependent VIL catabolism was required for wild type growth, and that Bat1 was unable to compensate lack of Bat2 (Table 2; Figure 1B, CEN). Accordingly, single *bat2Δ* and double mutants recovered wild type growth when transformed with a centromeric plasmid harboring *BAT2*, complementation failed with plasmids carrying *BAT1* (Figure 1B, CEN *vs.* CEN *BAT1* and CEN *BAT2*). Since on glucose-ammonium-VIL the double and single *bat2Δ* mutants showed growth rates which were equivalent to those displayed by the wild type strain, it can be concluded that in glucose VIL catabolism fulfills nitrogen requirements.

These results indicate that Bat2 has a prominent role in VIL catabolism, while Bat1 catabolic role is only evidenced in a *bat2Δ* genetic background. The fact that as Figure 2 shows, Bat1 mitochondrial localization is conserved in the presence of VIL as sole nitrogen source indicates that Bat1 catabolic character is exerted in this compartment. It could be proposed that under these conditions VIL accumulation in the mitochondria, would enhance Bat1 catabolic character.

Above presented results indicate that in a wild type strain Bat1 displays a biosynthetic character while Bat2 has a prominent catabolic role.

### *KlBAT1* does not complement *bat2Δ* mutant strains

To analyze whether the *Kl*Bat1 enzyme was able to replace Bat1 or Bat2 in *S. cerevisiae*, a monocopy plasmid harboring the *KlBAT1* gene was independently transformed in both single mutants *bat1Δ* and *bat2Δ* and in the double mutant *bat1Δ bat2Δ*. Constructions were prepared in order to promote *KlBAT1* expression from either its own promoter or by the heterologous *BAT1* or *BAT2* promoters. When grown on ammonium-glucose the *bat1Δ* mutant harboring *KlBAT1* on a monocopy plasmid attained wild type growth regardless of the promoter used to drive its expression (Figure 1A). In the case of the *bat1Δ bat2Δ* double mutant, the presence of *KlBAT1* only restored 72% of wild type growth (Figure 1A); indicating that *Kl*Bat1 could only partially substitute simultaneous lack of Bat1 and Bat2. When growing on VIL-glucose neither the *bat2Δ* nor the double mutant attained wild type growth when *KlBAT1* expression was driven from the *BAT1*, *BAT2* or *KlBAT1* promoters (Figure 1B), although higher growth rates were attained with P*_BAT2_*-*KlBAT1* or P*_KlBAT1_*-*KlBAT1*, suggesting that a promoter-dependent effect could enhance *KlBAT1* capacity to complement lack of Bat2. It could be possible that either the *Kl*Bat1 heterologous enzyme has peculiar kinetic properties that do not allow full *bat2Δ* complementation, or that the differential subcellular localization of *Kl*Bat1 and Bat2, could hamper *bat2Δ* complementation, since as mentioned earlier, Bat2 is a cytosolic enzyme and although localization of *Kl*Bat1 has not been experimentally determined, an *in silico* analysis using Mitoprot and SignalP databases suggests that *Kl*Bat1 is located in the mitochondria.

### *KlBAT1* has a biosynthetic-like expression profile

**Figure 3:**
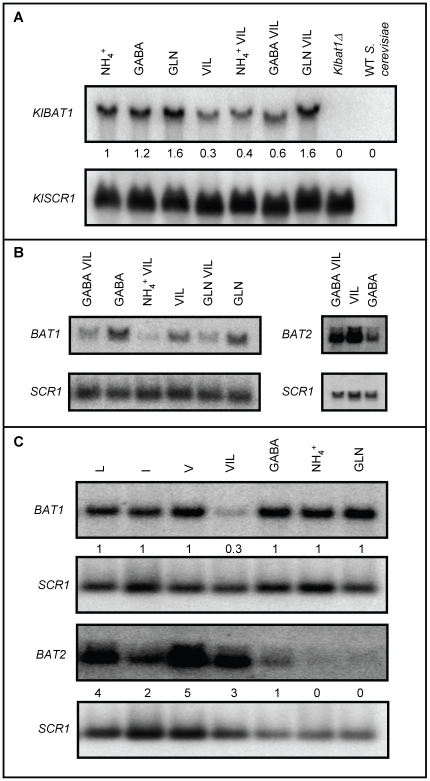
*Saccharomyces cerevisiae BAT1* and *KlBAT1* expression is repressed by VIL. Northern analysis was carried out on total RNA obtained from *K. lactis* 155 (wild type) and CLA34 (*Klbat1*Δ) strains (A), and *S. cerevisiae* strain CLA1-2 (wild type B, C). Strains were grown on 2% glucose with either valine (V) (150 mg/l), leucine (L) (100 mg/l), isoleucine (I) (30 mg/l), γ-aminobutiric acid (GABA 7 mM ), γ-aminobutiric acid+VIL (GABA VIL),VIL (valine+isoleucine+leucine), NH_4_ (40 mM NH_4_ SO_2_), NH_4_ VIL (40mM NH_4_ SO_2_+VIL), glutamine (GLN 7mM), glutamine+VIL (GLN VIL), as nitrogen sources. Filters were sequentially probed with the *BAT1*, *BAT2*, *KlBAT1*- specific PCR products described in experimental procedures and a *Bam*H1-*Hind*III 1500 bp *ACT1* DNA or an SCR 400bp PCR fragment as loading controls. Numbers indicate relative expression as compared to WT grown on ammonium-glucose. Four biological replicates were carried out, representative results are shown.

Total RNA was prepared from *K. lactis* wild type strain grown on glucose as carbon source with various nitrogen sources. It was found that *KlBAT1* expression profile was that expected for a biosynthetic enzyme. Steady state mRNA levels were similar in total RNA samples obtained from cultures grown on either repressive (glutamine) or non-repressive nitrogen sources (GABA), indicating that the quality of the nitrogen source had no effect on *KlBAT1* expression (Figure 3A). However, expression was repressed in total RNA samples obtained from cultures grown in the presence of VIL as sole nitrogen source, or when combined with additional nitrogen sources such as ammonium or GABA, as compared to that found in the absence of VIL (Figure 3A). Worth of mention is the fact that VIL repression was not observed in glutamine, suggesting that this amino acid could hinder VIL transport, thus resulting in a low intracellular accumulation of these amino acids.

### *BAT1* and *BAT2* show divergent expression profiles

**Figure 4:**
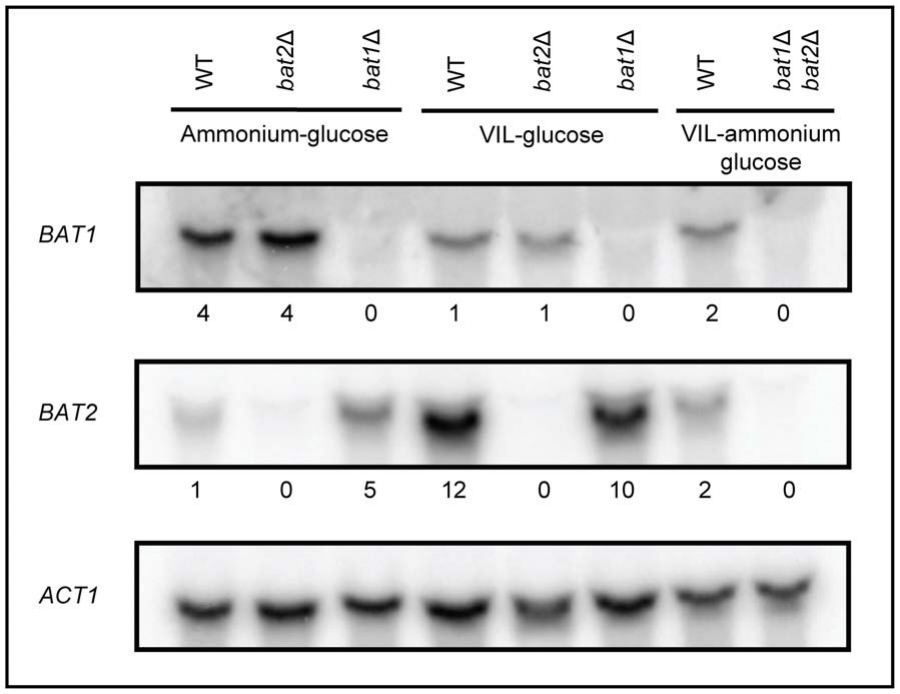
*S. cerevisiae BAT1* and *BAT2* display divergent expression profile. Northern analysis was carried out on total RNA obtained from *S. cerevisiae* strains CLA1-2 (wild type), CLA31 (*bat1Δ BAT2*) and CLA32 (*BAT1 bat2Δ*). Strains were grown on 2% glucose with either 40mM NH_4_ SO_2_, VIL (150 mg/l, 100 mg/l or 30 mg/l of valine (V), leucine (L) or isoleucine (I) respectively or NH_4_ SO_2_+VIL as nitrogen sources. Filters were sequentially probed with a 1500 bp *BAT1* fragment, a 1450 bp *BAT2* and a *Bam*H1-*Hind*III 1500 bp *ACT1* DNA fragment as loading control. Numbers indicate relative expression as compared to: Lane 1 the WT grown on glucose VIL, Lane 2 WT grown on glucose NH_4_. Four biological replicates were performed, and representative results are shown.

To analyze whether the apparent divergence in Bat1 and Bat2 metabolic roles, was correlated with the expression profile of their encoding genes, Northern analysis was carried out. It was found that as well as *KlBAT1*, *BAT1* was mainly expressed on ammonium-glucose exponential cultures (biosynthetic conditions), and repressed in the presence of VIL. *BAT1* expression was not influenced by the quality of the nitrogen source, and VIL repression was observed on either repressive or non-repressive nitrogen sources (Figure 3B and 3C). Conversely, *BAT2* showed a classic catabolic expression profile; responding to the quality of the nitrogen source; down-regulated in the presence of repressive nitrogen sources (glutamine) and derepressed in secondary non-repressive nitrogen sources such as GABA (Figure 3B and 3C). *BAT2* expression was twelve-fold increased when total RNA was obtained from cultures in which VIL was provided as sole nitrogen source (catabolic conditions), as compared to that found when RNA was prepared from on ammonium-glucose cultures (Figure 4). *BAT2* expression was also induced in a *bat1Δ* genetic background. Under derepressed conditions (GABA), the addition of the three branched chain amino acids, had a positive effect further inducing *BAT2* expression (Figure 3B).

### *Kl*Bat1 enzymatic activity displays a biosynthetic character

**Figure 5:**
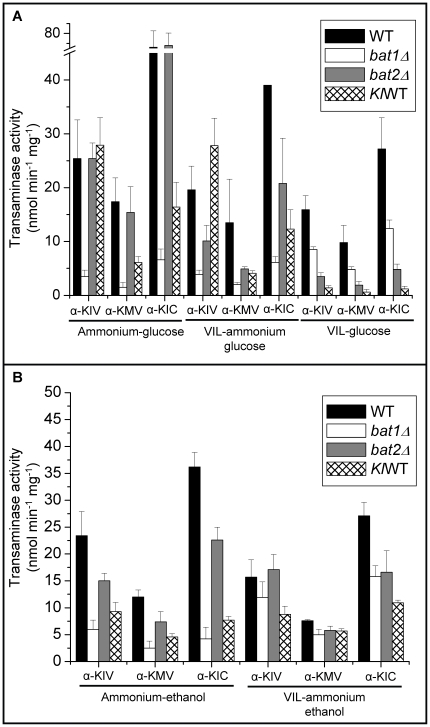
Branched chain aminotransferase activity. *S. cerevisiae* wild type, *bat1Δ*, *bat2Δ* and *K. lactis* wt (*Kl*WT) strains were grown on glucose (A) or ethanol (B) as carbon source and ammonium or VIL as nitrogen sources. Aminotransferase activity was determined in cell free extracts as indicated in Materials and methods using α-ketoisovalerate (α-KIV), α-ketoisocaproate (α-KIC) or α-ketomethylvalerate (α-KMV) as substrates. Transaminase activity is reported as nmol mg/l min^−1^. Values are presented as mean from at least three measurements ± S. D.

*Kl*Bat1 activity was determined in extracts obtained from cultures grown under biosynthetic and catabolic conditions (Figure 5A). Activity was similar in extracts obtained from ammonium-glucose with or without VIL (Figure 1A), indicating that the observed repression of *KlBAT1* expression on VIL-ammonium did not result in decreased enzymatic activity. When α-ketoisovalerate (α-KIV) was used as substrate, activity was nearly two-fold higher to that found with α-ketoisocaproate (α-KIC), indicating differential kinetic properties for these substrates. Lowest activity was detected on α-ketomethylvalerate (α-KMV). In extracts obtained from VIL-glucose (catabolic conditions), *Kl*Bat1 activity was at least ten-fold lower than that observed on ammonium-glucose (biosynthetic conditions), confirming *KlBAT1* expression profile (Figure 5A) and indicating that *Kl*Bat1 has a pronounced biosynthetic character.

### Bat1 and Bat2 enzymatic activity is consistent with *BAT1* and *BAT2* expression profile

BCAT enzymatic activity was determined in extracts obtained from the wild type and the single *bat1Δ* and *bat2Δ* mutants. As Figure 5A shows, activity determined on ammonium-glucose (biosynthetic conditions) was higher in the *bat2Δ* (*BAT1*) mutant, as compared to that found in the *bat1Δ* (*BAT2*) mutant strain, although in the presence of VIL-ammonium Bat1 activity decreased, it was however higher than that observed for Bat2. Conversely, in the presence of VIL as sole nitrogen source (catabolic conditions) Bat2 activity was higher than that observed for Bat1. These results are in agreement with expression profile observed for either *BAT1* or *BAT2*, which indicate that *BAT1* expression is VIL repressed while that of *BAT2* is induced in VIL as sole nitrogen source (Figure 4). These results support the proposition that BCAT biosynthetic and catabolic roles have been distributed between the two paralogous enzymes. Bat1 and Bat2 have diverged acquiring a biosynthetic or catabolic character, respectively. However, since only the double *bat1Δ bat2Δ* is a full VIL auxotroph unable to utilize VIL as sole nitrogen source, it can be concluded that the biosynthetic and catabolic roles of these enzymes is partially redundant. Highest activities were detected when α-KIC (leucine) was provided as substrate, as compared to that for α-KIV (valine) or α-KMV (isoleucine), indicating differential kinetic properties for the various substrates that could reflect differential substrate affinity.

### *BAT1* and *BAT2* show differential expression profiles and enzymatic activity under respiratory conditions

The collection of single and double *bat1Δ* and *bat2Δ* mutants was grown on ethanol as carbon source and ammonium as nitrogen source. Under this condition, growth of the double *bat1Δ bat2Δ* mutant was completely impaired, even in the presence of the three amino acids, indicating that VIL catabolism is compelling for growth in the presence of non-fermentable carbon sources, even on ammonium as nitrogen source (Table 3). Conversely, *Klbat1Δ* mutant was able to sustain growth in media supplemented with VIL-ammonium-ethanol, indicating that as opposed to that observed in *S. cerevisiae*, VIL catabolism is not necessary to achieve growth in the presence of ethanol as sole carbon source and ammonium as nitrogen source, underscoring the fact that *K. lactis* has a more efficient ethanol catabolism [15]. Accordingly, the *K. lactis* wild type strain achieved a higher growth rate than that attained by the *S. cerevisiae* wild type CLA1-2 strain when grown on ethanol as sole carbon source (0.21 *vs.* 0.12 h^−1^). On ammonium-ethanol, the *bat2Δ* mutant and the wild type strain, showed equivalent growth rates, while *bat1Δ* showed a slightly decreased growth rate as compared to the wild type strain, which was alleviated in the presence of the three amino acids, thus confirming Bat1 biosynthetic role. As expected, single mutants and wild type strain showed equivalent growth rates in the presence of the three amino acids. When ethanol was provided as the sole carbon source and the branched amino acids as sole nitrogen source neither the *S. cerevisiae* wild type strain nor the single or double mutants grew, indicating that these amino acids are poorly catabolized and thus unable to allow growth under these conditions (Table 3). Conversely, on VIL-ethanol, *K. lactis* wild type and the *Klbat1Δ* mutant complemented with the centromeric plasmid harboring the *KlBAT1* gene with its native promoter sequence, were able to sustain growth, confirming *Kl*Bat1 catabolic character (Table 3).

**Table 3:** Growth phenotypes of single and double *bat1Δ* and *bat2Δ* mutants.

	Relative growth^a^ (%)		
	Ethanol		
Strain	NH_4_^+^	NH_4_^+^ VIL	VIL
CLA1-2 (WT)	100	100	0
CLA31 (*bat1Δ*)	87	93	0
CLA32 (*bat2Δ*)	100	94	0
CLA33 (*bat1Δ bat2Δ*)	0	0	0
*Kl*WT	100	100	71
CLA34 (*Klbat1Δ*)	0	64	0
CLA34 (CEN *KlBAT1*)	90	100	85

a Values are shown relative to growth rate of the wild type strain (0.20 h^−1^ and 0.11 h^−1^ on NH_4_^+^ and amino acids, respectively); and represent the means from three independent experiments (variation was always ≤10%).

b Amino acids were supplemented at a concentration of 150 mg/l, 100 mg/l or 30 mg/l of valine (V), leucine (L) or isoleucine (I) respectively.

**Figure 6:**
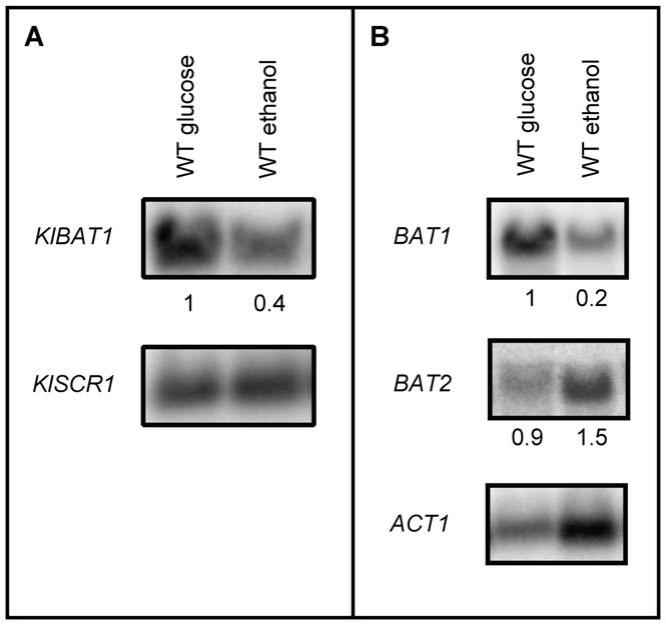
*BAT1* and *KlBAT1* expression is repressed under respiratory conditions. Northern analysis was carried out with total RNA samples obtained from *S. cerevisiae* WT, *bat1Δ BAT2* and *BAT1 bat2Δ* and *K. lactis* WT cultures grown on ammonium-glucose or ammonium-ethanol to mid exponential growth phase. Filters were sequentially probed with the *BAT1*, *BAT2*, *KlBAT1*- specific PCR products described in experimental procedures and a *Bam*H1-*Hind*III 1500 bp *ACT1* DNA or an *SCR1* 400bp PCR fragment as loading controls. Numbers indicate relative expression as compared to the WT strains grown on ammonium-glucose. Four biological replicates were performed, and representative results are shown.

Northern analysis performed with extracts obtained from ammonium-ethanol grown cultures, showed that *BAT1* and *BAT2* displayed opposed expression profiles; *BAT1* expression was five-fold repressed, while that of *BAT2* was two-fold increased on ethanol as compared to those found on glucose. *KlBAT1* showed a similar expression pattern to that of *BAT1*, since its expression was decreased on ethanol as compared to glucose (Figure 6A and 6B).

In extracts prepared from ammonium-ethanol, Bat1 activity (*bat2Δ*) decreased when either one of the three α-ketoacids were used as substrates, as compared to that found on glucose ammonium, while that of Bat2 (*bat1Δ*) was nearly two-fold increased as compared to that found on glucose (Figure 5A and 5B ). These results suggest that under respiratory conditions, Bat1-dependent α-ketoacid utilization would be diminished; avoiding increased carbon flux being channeled to VIL biosynthesis, while enhanced Bat2 activity would increase VIL utilization, favoring *S. cerevisiae* capacity to grow under respiratory conditions. On ammonium ethanol VIL, Bat1 activity was equivalent to that found without VIL, and Bat2 activity was two or three-fold increased as compared to ammonium ethanol (Figure 5B), thus under VIL-ammonium-ethanol, Bat1 and Bat2 showed equivalent enzymatic activities, indicating that both enzymes could equally contribute to VIL catabolism, in fact as Table 3 shows, growth rate of either *bat1Δ* or *bat2Δ* is similar, and growth is only impaired in the double mutant. It can be concluded that under respiratory conditions, Bat1 and Bat2 play partially biosynthetic redundant roles, and redundant catabolic roles.

As well as for Bat1, *Kl*Bat1 activity was three-fold decreased in extracts prepared from ammonium-ethanol grown cultures, as compared to those found on ammonium-glucose (Figure 5A and 5B) in agreement with the expression profile, and as well as for Bat1, addition of VIL to ammonium-ethanol growth medium did not affect activity, suggesting that under respiratory conditions, biosynthesis is decreased and catabolism is triggered, thus favoring an equilibrated consumption and synthesis of α-ketoacids.

## Discussion

This study addresses the question of whether the biosynthetic and catabolic roles played by the ancestral-like *KlBAT1* encoded aminotransferase present in *K. lactis*, have been distributed in the paralogous *BAT1* and *BAT2* orthologous genes present in *S. cerevisiae* and whether this subfunctionalization has improved branched chain amino acid metabolism constituting an adaptation to facultative metabolism.

### *BAT1* and *BAT2* divergent expression profiles and differential subcellular localization contribute to Bat1 and Bat2 functional diversification

Presented results show that the *KlBAT1* orthologue codifies a bifunctional enzyme able to carry out BCAAs biosynthesis and catabolism and that this capacity has been distributed in the *BAT1* and *BAT2 S. cerevisiae* paralogous pair.

Under respiro-fermentative conditions *BAT1* and *BAT2* divergent expression has contributed to emphasize the biosynthetic function of Bat1 and the catabolic function of Bat2. The observation that *BAT1* expression is four-fold higher than that of *BAT2* when cells are grown on glucose ammonia, and that *BAT2* expression is twelve-fold increased in the presence of a non-repressive nitrogen source and further enhanced when VIL is present as sole nitrogen source as compared to that found on ammonium, supports this proposition. Expression differences impact BCAT activity, in the presence of ammonium-glucose Bat1 activity is higher than that of Bat2 improving Bat1 biosynthetic capacity. Conversely, in the presence of glucose as carbon source and VIL as sole nitrogen source, Bat2 activity is enhanced, thus favoring its catabolic role. Bat1 has a limited catabolic character, which is most evident in the double *bat1Δ bat2Δ* mutant, which is completely unable to utilize VIL as nitrogen source. The fact that *bat1Δ* is a valine braditroph indicates that Bat2 valine biosynthetic capacity is limited or that the cytosolic valine pool is unable to enter the mitochondria, and thus Bat1 constitutes a committed step to synthesize the valine mitochondrial pool. These observations underscore the role of differential localization in Bat2 and Bat1 divergence and put forward the possibility that restricted biosynthesis or transport of the cytosolic generated valine pool to the mitochondria could act as positive selection determining *BAT1* retention and Bat1 mitochondrial localization.

### *BAT1* and *BAT2* retention constitutes an adaptation to facultative metabolism

*BAT1* expression is higher under fermento-respiratory conditions as compared to that detected under respiratory metabolism, while *BAT2* expression is increased under respiratory conditions. Reduced *BAT1* expression under respiratory conditions could contribute to decreased metabolite flow to amino acid biosynthesis favoring energy yielding pathways. Conversely, increased Bat1 activity under fermento-respiratory conditions, would not hinder energy provision, since under these conditions decreased intermediate flow through the tricarboxylic cycle does not hamper energy provision, constituting an adaptation to facultative metabolism.

Under fermentative or respiratory conditions, *Klbat1Δ* mutant is able to grow in the presence of the three BCAAs, indicating that catabolism is not required for growth, however the *bat1Δ bat2Δ* mutant of *S. cerevisiae* is unable to grow under these conditions suggesting that BCAAs catabolism is compelling. It could be considered that retention of the two paralogues in *S. cerevisiae* has led to efficient VIL degradation. In this regard it has been found that BCAA catabolism through Bat1 and Bat2 plays a major role in the production of higher alcohols such as isobutanol, active amyl alcohol and isoamyl alcohol, which have a great impact on beer smell and taste in either fermento-respiratory or respiratory conditions [12], [16]. Although incapacity to produce higher alcohols should not affect growth rate, the fact that either one of the two single mutants are unable to grow on ethanol-VIL, suggests that catabolism of these compounds in addition to the production of fusel alcohols could provide intermediates indispensable for growth under respiratory conditions. Since fusel alcohols are not further metabolized it could be considered that the main product contributed by BCAA catabolism could be oxidized NAD^+^ produced when fusel aldehydes are reduced to fusel alcohols through the Ehrlich pathway, however this possible role of the Ehrlich pathway remains to be analyzed [17]. Thus retention of *BAT1* and *BAT2* ensure BCAAs catabolism and growth under respiratory conditions in *S. cerevisiae*. This could have acted as a positive selection leading to *BAT1* and *BAT2* retention.

### Concluding remarks

Genetic redundancy is a major feature of virtually all species; duplication of functional genes constitutes a source of new or specialized functions of the encoded proteins. Duplicate genes that are retained either provide an increased dosage of the same product or go through a process of subfunctionalization, during which both copies of the gene lose a subset of their ancestral functions, while acquiring new properties [7], [8], [18].

*BAT1* and *BAT2* retention and acquisition of divergent expression profiles, warrants amino acid and α-ketoacid provision under fermento-respiratory and respiratory conditions. In addition, distribution of the biosynthetic and catabolic character of the BCAT in two isozymes could contribute to the avoidance of futile cycles since the independent regulation of each gene determines the presence of the pertinent isozymes under either biosynthetic or catabolic physiological conditions. The divergent physiological role played by Bat1 and Bat2 is further enhanced through differential localization; each enzyme is located in the compartment in which the pertinent substrates are produced. The fact that *K*lBat1 is mainly biosynthetic and its catabolic role is only exerted when VIL is added to the medium excludes the operation of futile cycles.

It has been proposed that the specialization of the *GDH1*- and *GDH3*-encoded NADP-dependent glutamate dehydrogenases and the *LYS20-LYS21*-encoded homocitrate synthases could result in the formation of hetero-oligomeric structures showing biochemical properties distinct from those displayed by the homo-oligomers, and which could play an important role under certain environmental conditions [4], [6]. Building up of Gdh1-Gdh3 or Lys20-Lys21 hetero-oligomeric isoforms is possible since both enzymes are located in the same subcellular compartment. For Bat1 and Bat2, constitution of hetero-oligomeric isoforms would be hindered by differential localization. Since in many cases oligomerization domains are conserved in paralogous proteins, differential subcellular localization would avoid hetero-oligomerization, preventing the formation of hybrid isozymes whose biological activity could be hindered. The fact that the bifunctional role played by the ancestral-like *Kl*Bat1 has been distributed in Bat1 and Bat2, which could be presumed to be oligomeric enzymes [19]–[21] suggests that for this case, formation of hetero-oligomeric forms could hinder their biological activity, underscoring the role of enzyme relocalization on the functional diversification of duplicate genes.

This study provides an example indicating that the improvement of the functions carried out by a bifunctional gene product can be achieved through gene duplication and further subfunctionalization as has been shown to be the case for the genetic switch controlling the yeast galactose utilization pathway. In *S. cerevisiae*, two paralogous genes encode the Gal3 co-inducer and the *GAL1*-encoded galactokinase, which in *K. lactis* are contained in a single bifunctional ancestral-like gene [22].

Finally and worth mentioning is the existence in *S. cerevisiae* genome of three pairs of duplicated genes respectively encoding piruvate, aspartate and aromatic aminotransferases (*ALT1-ALT2, AAT1-AAT2* and *ARO8-ARO9*). *ALT1-ALT2* belong to the duplicated blocks acquired after the WGD event, while *AAT1-AAT2* and *ARO8-ARO9* correspond to independent duplication events. Thus, the described duplication and further diversification of *BAT1-BAT2* may be representative of a general mechanism through which *S. cerevisiae* has improved amino acid metabolism.

## Materials and Methods

### Strains

Table 4 describes the characteristics of the strains used in the present work. Independent *bat1Δ* and *bat2Δ* derivatives of the CLA1-2 (*ura3 leu2::LEU2*) [4] were obtained using the PCR-based gene replacement protocol described by Wach *et al*. [23], with *kanMX4* as a marker. Four deoxyoligonucleotides were designed respectively based on the *BAT1* (M1 and M2) or *BAT2* (M3 and M4) nucleotide sequences and that of the multiple cloning site present in the pFA6a vector [23] (oligonucleotides used for this study are described in Table S1). QIAGEN purified pFA6a DNA was used as template for PCR amplification in a Stratagene Robocycler 40 using standard amplification protocols. The obtained 1584-bp and 1586-bp PCR products respectively harboring *BAT1* or *BAT2* sequences were gel-purified and used to transform strain CLA1-2, generating strains CLA31 (*bat1Δ::kanMX4 BAT2 ura3 leu2::LEU2*) and CLA32 (*BAT1 bat2Δ::kanMX4 ura3 leu2::LEU2*).

**Table 4:** Strains used in this work.

Strain	Relevant phenotype	Source
CLA1-2	*MATα BAT1 BAT2 ura3 leu2::LEU2*	[4]
CLA31	*MATα bat1Δ::kanMX4 BAT2 ura3 leu2::LEU2*	This study
CLA32	*MATα BAT1 bat2Δ::kanMX4 ura3 leu2::LEU2*	This study
CLA33	*MATα bat1Δ::NAT bat2Δ::kanMX4 ura3 leu2::LEU2*	This study
*Kluyveromyces lactis* 155	*MATα ade2 his3 ura3*	[37]
CLA34	*K. lactis* 155 *Klbat1Δ::kanMX4*	This study

A CLA1-2 *bat1Δ bat2Δ* derivative (CLA33) was isolated from a nourseothricin resistant derivative of the CLA31 *bat1Δ* single mutant obtained by transforming this strain with p4339 *Eco*RI digested plasmid, which bears a copy of clonNAT gene [23], that replaces the *kanMX4* module by homologous recombination, generating strain CLA31-a (*bat1Δ::natMX4 BAT2 ura3 leu2::LEU2*). A *bat2Δ* derivative was generated from CLA31-a, as described above.

To obtain a *Klbat1Δ* mutant, from the *Kluyveromyces lactis* (*K. lactis*) orthologous *BAT1/BAT2* gene (KLLA0A10307g; *KlBAT1* in this study) *KlBAT1* was replaced by homologous recombination using a module containing the *kanMX4* cassette flanked by 95 bp of 5′ UTR (−105 to −10) and 101 bp of 3′ UTR (+1228 to +1329) sequences of *KlBAT1* gene. The module was amplified from pFA6a plasmid by using deoxyoligonucleotides M5 and M6 (Table S1). The PCR product was purified by using the Wizard SV Gel and PCR Clean-Up System (PROMEGA) and used as template for a second PCR in order to extend the homologous recombination regions. Second PCR was amplified with oligonucleotides M7 and M8 (Table S1). Yeasts were transformed by the method described by Ito *et al*. [24]. Transformants were selected for either G418 resistance (200 mg/l; Life Technologies, Inc.), or nourseothricin resistance (100 mg/l; Werner BioAgents) or both, on yeast extract-peptone-dextrose (YPD)-rich medium.

### Growth conditions

Strains were routinely grown on MM containing salts, trace elements, and vitamins following the formula of yeast nitrogen base (Difco). Glucose (2%, w/v) or ethanol (2%, w/v) was used as a carbon source, and 40 mM ammonium sulfate was used as a nitrogen source. Valine (150 mg/l), leucine (100 mg/l), isoleucine (30 mg/l), adenine (20 mg/l), histidine (20 mg/l) or uracile (20 mg/l) were added at the indicated final concentrations when required. 7 mM glutamine or GABA were supplemented when needed. Cells were incubated at 30°C with shaking (250 rpm).

### Construction of low copy number plasmids bearing *BAT1*, *BAT2* or *KlBAT1* genes

All standard molecular biology techniques were followed as described by Sambrook *et al*. [25]. *BAT1* or *BAT2* were PCR-amplified together with their 5′ promoter sequence and cloned into the pRS416 (*CEN6 ARSH4 URA3*) low-copy-number plasmid [26], [27]. For *BAT1*, a 2315 bp region between −1080 from the start codon and +293 from the stop codon was amplified with deoxyoligonucleotides M9 and M10 (Table S1) generating plasmids pRS416-*BAT1* and pRS426-*BAT1*. For *BAT2*, a 1681 bp region between −471 from the start codon and +79 from the stop codon was amplified with deoxyoligonucleotides M11 and M12 generating plasmids pRS416-*BAT2* and pRS426-*BAT2*. For *KlBAT1* a 1791 bp region between −500 from the start codon and +67 from the stop codon was amplified with deoxyoligonucleotides M13 and M14 (Table S1) The PCR fragment was then cloned into YEpKD352 (pKD1 ori *URA3*) plasmid (kindly provided by Dr. Roberto Coria) or pRS416 (*CEN6 ARSH4 URA3*) plasmid generating YEpKD352-*KlBAT1* and pRS416-*KlBAT1* respectively. DNA sequencing was carried out, using the T3/T7 priming sites of pRS316 and pRS426, at the Unidad de Biología Molecular, Instituto de Fisiología Celular, Universidad Nacional Autónoma de México (UNAM). Plasmids were subsequently transformed into CLA1-2 and isogenic *bat1Δ* and *bat2Δ* single mutants and *bat1Δ bat2Δ* double mutant or *K. lactis* wild type strain and *Klbat1Δ* mutant.

### Construction of *BAT1* and *BAT2* chimerical fusion plasmids

Fusions containing either the *BAT1* promoter and the *KlBAT1* coding sequence or the *BAT2* promoter and the *KlBAT1* coding sequence were generated by overlapping PCR amplification. For this purpose, primers M9 and M15 were used to obtain a 1092 bp product corresponding to the *BAT1* promoter sequence and the first 29 bp of the *KlBAT1* coding sequence; this was overlapped with the 1303 bp product of primers M16 and M14, which included the complete *KlBAT1* coding sequence. Similarly, primers M11 and M17 were used to obtain a 483 bp product corresponding to the *BAT2* promoter sequence, together with the first 29 bp of the *KlBAT1* coding sequence, and overlapped with the 1303-bp product of primers M16 and M14, which included the complete *KlBAT1* coding sequence. The modules obtained were cloned into the pRS416 plasmid, thus generating pRS416 P*_BAT1_*-*KlBAT1* and pRS416 P*_BAT2_*-*KlBAT1* plasmids. These plasmids were subsequently transformed into the CLA1-2 and isogenic *bat1Δ* and *bat2Δ* single mutants and *bat1Δ bat2Δ* double mutant.

### Construction of *BAT1* and *BAT2* β-galactosidase fusion plasmids

Transcriptional fusions of *BAT1* or *BAT2* promoters to the coding region of *lacZ* gene of *Escherichia coli* were generated by cloning the promoter regions into the YEp353 (2µ ori *URA3*) plasmid [28]. M9 and M18 deoxyoligonucleotides were used to amplify a 1122 bp sequence corresponding to the *BAT1* promoter and M11 and M19 deoxyoligonucleotides were used to amplify a 510 bp region corresponding to the *BAT2* promoter. Plasmids generated were YEp353 P*_BAT1_* and YEp353 P*_BAT2_*. These plasmids were transformed in CLA1-2 wild type strain.

### Construction of *BAT1* and *BAT2* yECitrine tagged mutants

*BAT1*-yCE and BAT2-yCE were prepared as described by Longtine *et al.* [29]. Two pairs of oligonucleotides were designed, based on either the *BAT1* (M20-M21) or *BAT2* (M22-M23) coding sequence and that of pKT175 deoxyoligonucleotides were used to amplify four PCR fragments, plasmids generated were transformed into the BY4741 yeast strain (Table 4). yECitrine-fusion constructs on the carboxy-end of either *BAT1* or *BAT2* was carried out as previously described [29], and PCR confirmed.

### Fluorescent microscopy

Bat1-yECitrine and Bat2-yECitrine tagged strains were used to asses these proteins subcellular localization through confocal microscopy. To confirm mitochondrial localization the strain Bat1-yECitrine was stained with Mito-Tracker Red CMXRos (Molecular Probes) according to manufacturers specifications. Co-localization between the Mito-Tracker and yECitrine was determined through sequential imaging. Confocal images were obtained using a FluoView FV1000 laser confocal system (Olympus) attached/interfaced to an Olympus IX81 inverted light microscope with a 60× oil-immersion objective (UPLSAPO 60× O NA:1.35), zoom ×20.0 and 3.5 µm of confocal aperture. The excitation and emission settings were as follows: yECitrine excitation at 488 nm; emission 520 nm BF 500 nm range 30 nm; Mito-Tracker excitation 543 nm; emission 598 nm, BF 555 nm range 100 nm. The images were collected in a sequential mode z-stack (5µm/slice) using a Kalman integration mode. The subsequent image processing was carried with Olympus FluoView FV1000 (version 1.7) software.

### Cell extract preparation

Cell extracts were prepared using a modified protocol from Rigaut *et al.* [30] and the NCRR Yeast Resource Center. Briefly, a 2 l yeast culture was grown to exponential phase in YPD, cells were collected by centrifugation at 4°C, pellets were washed with bi-distilled cold water and then with cold NP-40 buffer (Na_2_HPO_4_ 15 mM, NaH_2_PO_4_-H_2_O 10 mM, NP-40 1%, NaCl 150 mM, EDTA 2 mM, NaF 80 mM, Na_3_VO_4_ 0.1 mM, DTT 1 mM, BSA 0.1%, PMSF 1mM, pH 7.2). Cells are collected by centrifugation, suspended in 15–20 ml of NP-40 buffer and transferred to the 50 ml chamber of a bead beater. The same volume of glass beads was added to the suspended cells. Cells were then lysed in an iced bath with 7 cycles of 1 min on/1 min off, with a 5 min off period half-way through. Lysate was transferred to 50 ml Falcon tubes and clarified at 3,000 rpm for 10 min in a refrigerated centrifuge. The supernatant was collected and enzymatic activity was determined immediately.

### Branch chain aminotransferase enzymatic assay and protein determination

A previously described assay [31], coupled branch chain aminotransferase activity to NAD(P)H oxidation catalyzed by NAD(P)H Glutamate Dehydrogenase (GDH). However, under our experimental conditions NADP-GDH was able to use the branched-chain α-ketoacids as substrates, thus uncoupling the NAD(P)H oxidation from the branched-amino acid transaminase activity. An alternative method to measure branched-amino acid transaminase activity using the multienzyme α-ketoglutarate dehydrogenase complex from porcine heart (α-KGDH), was developed. Bat1 and Bat2 enzymes use the branched-chain α-ketoacids (BCKA) and glutamic acid as substrates to produce branched chain amino acids (BCAA) and α-ketoglutarate (α-KG), using pyridoxal 5′-phosphate (PP) as cofactor. Then, the (α-KG) produced can be used as substrate, along with Coenzyme A (CoA) and NAD^+^ by α-KGDH producing succinyl-CoA and CO_2_ thus reducing the NAD^+^ to NADH. NAD^+^ reduction was monitored measuring Absorbance at 340 nm along the time. The final volume of the assay was 1 ml containing 50 mM MOPS pH 7.1, 1 mM DTT, 0.1 mM CaCl_2_, 0.47 MgCl_2_, 1 mM thiamine pyrophosphate (C8754, SIGMA), 0.25 mM CoA (C4780, SIGMA), 0.25 mM pyridoxal 5′-phosphate (P9255, SIGMA), 0.25 U α-KGDH (K1502, SIGMA), 1 mM NAD^+^ (N7004, SIGMA), 5 mM potassium phosphate buffer pH 7.0 and variable concentrations of BCKA and glutamic acid (G1501, SIGMA). The reaction was started with the addition of crude cell extracts. All assays were carried out at 30°C in a Varian Cary 400 spectrophotometer with a 1 cm path length. BCKA used in this assay were: α-Ketoisocaproic acid sodium salt (α-KIC; K0629, SIGMA), DL-α-Keto-β-methylvaleric acid sodium salt (α-KMV; K7125, SIGMA) and Sodium methyl valerate (α-KIV; 151395, ICN).

### β-galactosidase activity determination

Soluble extracts were prepared by resuspending whole cells in the corresponding extraction buffer [32], cells were lysed with glass beads. β-galactosidase (β-Gal) activities were determined as previously described [33], [34]. Specific activity was expressed as nmoles of ο-nitrophenol produced per minute per milligram of protein. Protein was measured by the method of Lowry [35] using bovine serum albumin as a standard.

### Northern blot analysis

Northern analysis was carried out as described previously [34]. Total yeast RNA was prepared as described by Struhl & Davis [36] from exponentially grown cells (OD_600_ 0.4–0.6) or stationary grown cells (3–5 days) in 100 ml cultures. *BAT1*, *BAT2* and *KlBAT1* probes were amplified using M24 and M10, M25 and M26, and M16 and M27 deoxyoligonucleotides. *BAT1*, *BAT2* and *KlBAT1*. Probes include the whole coding region and promoter of each gene. Blots were scanned using the program ImageQuant 5.2 (Molecular Dynamics). Either a 473 bp *KlSCR1* fragment amplified on *K. lactis* genomic DNA preparation, using deoxyoligonucleotides M28 and M29 or a 1200 bp *ACT1* fragment were used as loading controls.

## Supporting Information

Table S1 Deoxyoligonucleotides used in this study.
(DOC)
