# Supplementary material for: Saccharomyces cerevisiae Bat1 and Bat2 Aminotransferases Have Functionally Diverged from the Ancestral-Like Kluyveromyces lactis Orthologous Enzyme
Source: PLoS One. 2011 Jan 18;6(1):e16099. doi: 10.1371/journal.pone.0016099 (PMC3022659; doi:10.1371/journal.pone.0016099)
Supplement: Table S1 — Deoxyoligonucleotides used in this study. (DOC) [file pone.0016099.s001.doc]

**Table S1.** Deoxyoligonucleotides used in this study

| **Name** | **Sequence** | **Application** |
| --- | --- | --- |
| M1 | 5’-TAA ATC ACC CTA TAA ACG CAA AAT CAG CTA GAA CCT TAG Ccg tac gct gca ggt cga c-3’ | *BAT1::kanMX4* deletion module, it comprises 40 bp of the *BAT1* 5’ UTR (-50 to-10) and 18 bp (lowercase) of the pFA6a-*kanMX4* sequence |
| M2 | 5’-CGT TTT TTT TTT TTG GGG GGG GAG GGG ATG TTT ACC TTa tcg atg aat tcg agc tcg-3’ | *BAT1::kanMX4* deletion module, contains 38 bp corresponding to the 3’ UTR (+1190 to +1228) of the *BAT1* sequence and 19 bp (lowercase) from the pFA6a-*kanMX4* sequence |
| M3 | 5’-ATT CCT TTG AGA AAT CCT TAA TAA AAA CAG ACT AAC TAC Tcg tac gct gca ggt cga c-3’ | *BAT2::kanMX4* deletion module, it comprises 40 bp of the 5’ UTR (-90 to -50) of *BAT2* sequence and 18 bp (lowercase) from the pFA6a-*kanMX4* sequence |
| M4 | 5’-GTT TTA TTC TTT TTA ACT TTT AAT TAC TTT ACG TAG CAA Tat cga tga att cga gct cg-3’ | *BAT2::kanMX4* deletion module, it contains 40 bp of *BAT2* 3’ UTR (+1140 to +1180) and 19-bp (lowercase) from the pFA6a-*kanMX4* sequence |
| M5 | 5’-CCA TCT GTA TCA TTT GTC CTA GCT ATT TCA TCC CTG GCA AGG cgt acg ctg cag gtc gac-3’ | *KlBAT1::kanMX4* deletion module, the pFA6a-*kanMX4* sequence is indicated (lowercase) |
| M6 | 5’-GCT ACA TAG TTG CTA GTG ACA CAT AAT CAC GGT AGG TTG TAT TTA GGa tcg atg aat tcg agc tcg-3’ | *KlBAT1::kanMX4* deletion module, the pFA6a-*kanMX4* sequence is indicated (lowercase) |
| M7 | 5’-GGC AGA TAA GAA GGG CAT TTG TCT TGT TTC TGT TCT TGT TTG GCC CGT TAT TGT CAC CAT CTG TAT CAT TTG-3’ | Extension of homologous recombination 5’ UTR in *KlBAT1::kanMX4* deletion module |
| M8 | 5’-GAG AGA ACA GTT TAT AAG AGG GAA TAG AGA AAC TAT TTG AGC TAC CTG ATC CGT GCT ACA TAG TTG CTA GTG-3’ | Extension of homologous recombination 3’ UTR in *KlBAT1::kanMX4* deletion module |
| M9 | 5’-GCG CGC GAA TTC CGT TTA AGG TTC CTA TTT TGA TAG TC-3’ | *BAT1* coding sequence and promoter region, an *Eco*RI restriction site was introduced (underlined sequence) |
| M10 | 5’-CAT GTA AAG TCC AGC GAG ATA CCT TGG C-3’ | *BAT1* coding sequence and promoter region |
| M11 | 5’-GCG CGC GAA TTC AAA CCT GCC TCT GAG GGT CAT -3’ | *BAT2* coding sequence and promoter region, an *Eco*RI restriction sequence was introduced (underlined sequence) |
| M12 | 5’-GCG CGC CTC GAG CCC CTT AAC TCA GTA GAA AAA ACG C-3’ | *BA12* coding sequence and promoter region, a *Xho*I restriction sequence was introduced (underlined sequence) |
| M13 | 5’-GCG CGC GAA TTC CGT TGA CAA GTG ACA ATG CAT GAC-3’ | *KlBAT1* coding sequence and promoter region, underlined sequence indicates *Eco*RI restriction site |
| M14 | 5’-GCG CGC GGA TCC GAG CTA CCT GAT CCG TGC TAC ATA G-3’ | *KlBAT1* coding sequence and promoter region, underlined sequence indicates *Bam*HI restriction site |
| M15 | 5’-gca caa ttc cgt agt cca atg tag ttc atG TTT TAG TAT GCT AAG GTT CTA GCT G-3’ | *BAT1* promoter, it contains 29 bp of the *KlBAT1* coding sequence (lowercase) |
| M16 | 5’-ATG AAC TAC ATT GGA CTA CGG AAT TGT GC-3’ | *KlBAT1* coding sequence |
| M17 | 5’-gca caa ttc cgt agt cca atg tag ttc atA TCG TTC TTA AAA CTC GTG GAG ATG C-3’ | *BAT2* promoter, it contains 29 bp of the *KlBAT1* coding sequence (lowercase) |
| M18 | 5’-GCG CGC GCT GCA GGA ATG TCT CTG CAA CAT GTT TTA GTA T-3’ | *BAT1* promoter for transcriptional fusion to *lacZ, Pst*I restriction sequence is underlined |
| M19 | 5’-GCG CGC GCT GCA GGG TGC CAA GGT CAT ATC G-3’ | *BAT2* promoter for transcriptional fusion to *lacZ, Pst*I restriction sequence is underlined |
| M20 | 5’-ACG GTA GAG TCA ATT ATG GTA ACT GGT CAA AAA CTG TTG CCG ACT TGA ACg gtg acg gtg ctg gtt ta-3’ | *BAT1* coding sequence for traductional fusion to yECitrine, pKT175 sequence is indicated (lowercase) |
| M21 | 5’-TTC TCG TTT TTT TTT TTT GGG GGG GGA GGG GAT GTT TAC CTT CAT TAT CAt cga tga att cga gct cg-3’ | *BAT1* coding sequence for traductional fusion to yECitrine, pKT175 sequence is indicated (lowercase) |
| M22 | 5’-ATG GCG AGA CTG AGC ATG GCA ATT GGT CAA GGG TTG TTA CTG ATT TGA ACg gtg acg gtg ctg gtt ta**-**3’ | *BAT2* coding sequence for traductional fusion to yECitrine, pKT175 sequence is indicated (lowercase) |
| M23 | 5’-AGT TTT ATT CTT TTT AAC TTT TAA TTA CTT TAC GTA GCA ATA GCG ATA CTt cga tga att cga gct cg**-**3’ | *BAT2* coding sequence for traductional fusion to yECitrine, pKT175 sequence is indicated (lowercase) |
| M24 | 5’-ATC AAT AGT AAG GCT CGC AAA CCG CCC-3’ | *BAT1* probe for Northern blot analysis |
| M25 | 5’-CAA ACA TCT TCG AAC GTG AAA ACC TGC CTC TGA GGG-3’ | *BAT2* probe for Northern blot analysis |
| M26 | 5’-GTC CTT GAT ACC GAT AGG CCA GC-3’ | *BAT2* probe for Northern blot analysis |
| M27 | 5’-ACG GCT TTG AAC CCA GTC TTG TAA TAT GG-3’ | *KlBAT1* probe for Northern blot analysis |
| M28 | 5’-GGA GTG TGA TGC TTG GAT ATG TGG CTC-3’ | *KlSCR1* probe for Northern blot analysis |
| M29 | 5’-CAG TGT ATG TCT GCC TCT GAA CCC AC-3’ | *KlSCR1* probe for Northern blot analysis |
